# Pseudocholinesterase activity in cerebrospinal fluid as a biomarker of solid central nervous system tumors in children

**DOI:** 10.3325/cmj.2013.54.429

**Published:** 2013-10

**Authors:** Lili Mikecin, Miljenko Križmarić, Jasminka Stepan Giljević, Miroslav Gjurašin, Josipa Kern, Jasna Leniček Krleža, Ljiljana Popović

**Affiliations:** 1 University Clinical Center Maribor, Pediatric Intensive Care Unit, Maribor, Slovenia; 2 Faculty of Medicine, University of Maribor, Faculty of Health Sciences University of Maribor, Maribor, Slovenia; 3 Department of Hematology and Oncology, Children's Hospital Zagreb, Zagreb, Croatia; 4 Department of Neurosurgery, Children's Hospital Zagreb, Zagreb, Croatia; 5 Department of Medical Statistics, Epidemiology and Medical Informatics, School of Public Health Andrija Štampar, University of Zagreb Medical School, Zagreb, Croatia; 6 Department for Clinical Chemistry, Children's Hospital Zagreb, Zagreb, Croatia; 7 Department for Anesthesiology, Reanimatology and Intensive Care, Children's Hospital Zagreb, Zagreb, Croatia

## Abstract

**Aim:**

To determine the activity of pseudocholinesterase (PChE) in cerebrospinal fluid (CSF) and serum in children with solid central nervous system (CNS) tumor and to assess whether PChE activity could be a valid biomarker for solid CNS tumors in children.

**Methods:**

The study and control group included 30 children each. Children in the study group had a solid CNS tumor, while those from the control group had never suffered from any tumor diseases. CSF and serum samples were collected from all participants and PChE activity was determined using the Ellman’s spectrophotometric method. PChE activity in CSF was shown as a cerebrospinal fluid/serum ratio expressed in percentage, ie, PChE CSF/serum ratio. Receiver operating characteristic (ROC) curve was used to assess whether PChE activity can be used as a biomarker for identifying children with solid CNS tumors.

**Results:**

Children with solid CNS tumor had significantly higher PChE activity in CSF and serum, as well as PChE CSF/serum ratio (*P* = 0.001). PChE CSF/serum ratio in the study group was 2.38% (interquartile range [IQR] 1.14-3.97) and 1.09% (IQR 0.95-1.45) in the control group. ROC curve analysis of PChE CSF/serum ratio resulted in an area under the curve (AUC) value of 0.76 (95% confidence interval [CI] 0.63-0.88) and a cut-off of 1.09. Twenty five of 29 patients with elevated PChE CSF/serum ratio had a tumor, corresponding to a sensitivity of 83% and a specificity of 53%.

**Conclusion:**

PChE CSF/serum ratio may be used as a test or biomarker with good sensitivity for solid CNS tumors in children.

Pediatric solid central nervous system (CNS) tumors are rarely detected at an early stage. The reasons for this include the tumor site, acute intracranial bleeding, unavailable imaging tests, inconsistent conduct of clinical diagnostic procedures, etc (1). CNS tumors account for 20% of all childhood cancers, and their incidence rates are rising (2). Treatment results are better if the disease is detected early, which requires sufficiently sensitive and specific diagnostic methods. This is why new biomarkers indicating the presence of CNS tumors are needed.

One of the biological materials used for biomarker testing is cerebrospinal fluid (CSF) (3). CSF washes the structures of the CNS including the tumors present there, enabling the detection of tumor proteins in the CSF. PChE has a role in tumorogenesis and oncogenesis and has been proven to be a good indicator of the presence of tumors (4-6). PChE in serum has already been recognized and confirmed as a good diagnostic and prognostic marker for head and neck tumors (7). Studies of PChE in tumor tissue showed that its concentration in some tumor samples was high, eg, in glioma tissue samples (4). In tissue samples, PChE has higher activity levels in astrocytomas than in meduloblastomas, neuromas, or meningeomas (4). It is included in the myelin metabolism of, eg, the corpus callosum, nervus opticus, and spinal cord. Subcortical white matter has much greater PChE activity than gray matter (8) and PChE activity decreases as we descend from the cerebrum toward the spinal cord. After blood removal, PChE activity is present in the vessels of the brain and it is higher in arteries than in veins (4)

PChE is an enzyme (EC 3.1.1.8) synthesized in the liver and distributed to body tissue and fluids. Cholinesterases (acetylcholinesterase – AChE and PChE) activity changes in an abnormal tissue metabolism and/or they become part of the oncogenic process (7). Cholinesterase genes are amplified, aberrant, and mutated in numerous types of human tumors. The PChE and AChE found in these tumors contain the peptide motif S/T-P-X-Z found in many cdc2 gene-dependent protein kinase substrates (5). Phosphorylation of cdc2-dependent protein kinases is a possible molecular mechanism that connects cholinesterases with tumor proliferation. Moreover, the inhibition or reduction of AChE, PChE, and some other enzymes especially with organophosphorous and perhaps carbamate poisons used as insecticides in home and local environment may result in the formation of brain tumors in children partially depending on the genetic polymorphisms that affect insecticide metabolism (9,10).

PChE activity in CSF in healthy people is rarely investigated, most often as a part of studies on specific diseases or anesthetics (11-13). Lumbar puncture is an invasive procedure and for ethical reasons the number of healthy participants in studies is always small, especially in case of children. For this reason the information about the normal values of PChE activity in CSF in relation to age at all ages is scarce. In adults, PChE activity in CSF ranges from 1/20 to 1/100 of the value of serum (13). In children, the values are age-dependent (14). PChE activity in serum is low at birth, which is followed by a sudden increase in activity over the next three weeks, with values exceeding those in adulthood. This remains unchanged until the age of three. Between three and six years, PChE activity is 30% above the adult level and it begins to slowly drop between the age of five and puberty, when it reaches the adulthood value, which remains relatively stable (14). In this study, we tested whether PChE could be used as a potential biomarker for solid CNS tumors in children.

## Patients and methods

The study was conducted at the Reference Center for Solid Tumors in Children of the Ministry of Health and Social Welfare in Croatia, Children’s Hospital Zagreb, Croatia, between October 1, 2007 and November 30, 2011. Ethical approval was received from the Ethics Committee of the Children’s Hospital Zagreb. Written consent was obtained for each child from their parents, who had been explained the study objectives and procedures verbally and in writing.

The number of study participants was limited to 60 because we had only 60 laboratory kit tests. We had 30 patients with solid CNS tumor and 30 patients who met the criteria for the control group. When all the available laboratory kit tests had been used we finished the study. There was a total of 60 participants aged between 2 months and 16 years. The study group comprised of 30 children with solid CNS tumors. Tumors were classified according to the 2007 World Health Organization classification (1,15). We divided the study group according to tumor classification and histopathological test result into gliomas (low-grade and high-grade), ependymomas, embryonal tumors, germ cell tumors, and craniopharyngiomas. In the study group 9 children had gliomas, 7 had low-grade gliomas, out of whom 1 had an anaplastic ganglioglioma, 2 had astrocytomas, and 4 had gliomas. In the high-grade glioma group, 2 children had glioblastomas. Five children had ependymomas, 14 embryonal tumors, 1 child had a germ cell tumor, and 1 child had craniopharyngioma. In the embryonal tumor groups, 12 children had meduloblastomas, 1 child had a primitive neuroectodermal tumor, and 1 child had an atypical teratoid rhabdoid tumor. All tumors primarily originated from the CNS.

The control group comprised 30 children who had never suffered from any tumor diseases. Eight children were hospitalized due to the management of febrile convulsion; after a neurotrauma without any sign of hemorrhaging into the CNS (7 children); for the replacement of the ventriculoperitoneal drain system as a result of growth in case of Arnold Chiari malformation (3 children) and Dandy Walker malformation (4 children); because of the congenital hydrocephalus (6 children); meningomyelocele (1 child); and syringomyelia (1 child). We chose these children because each child had normal neurological status or only chronic motor handicap; brain CT result showing only anatomical variations, and normal biochemical, cytological, and microbiological CSF test results. We did not choose random healthy children, since they do not have an indication for the lumbar puncture, which would interfere with the ethical principles.

Samples of blood and CSF (both 0.5 µL) were collected at the same time, only once from each child. The samples were immediately centrifuged and separated from erythrocytes, and then stored at -70°C. We determined PChE activity using the Ellman’s spectrophotometric method with a Careside TM cholinesterase test system (Careside Inc, Culver City, CA, USA) using p-hydroxybenzoil choline as a substrate, manufactured by Vitros DT Slides (16).

There was a difference in PChE activity between age groups, which was even more pronounced in girls. Moreover, previous measurements of PChE only in CSF showed high variability (13). This is why we used PChE CSF/serum ratio, presented in percentage, as a variable expected to be unaffected by age, sex, or tumor type/pathological developments.

Cytological evaluation (complete cell count), microbiological (bacteriological), and biochemical analysis (glucose in mmol/L, Cl in mmol/L, total protein in g/L, and albumin fraction in g/L) was performed on CSF obtained by lumbar puncture. All drugs taken by the participants at the time of sampling were noted.

### Statistical analysis

All analyses were performed using the IBM SPSS statistical package version 20.0 (IBM Corp., Armonk, NY, USA). As PChE levels were found not to be normally distributed, results are reported as median and interquartile ranges (IQR). In a skeletal box-and-whisker plot, the whiskers are drawn from the quartiles to the extreme values of the group. Normality was evaluated with the Kolmogorov-Smirnov and the Shapiro-Wilk test. Group comparisons were performed using the nonparametric Mann-Whitney U tests for continuous data and Fisher exact test for categorical data. As a nonparametric measure of statistical dependance between two variables we used Spearman's rank correlation coefficient. The significance level was set at *P* < 0.05. Receiver operator characteristic (ROC) curve analysis was used for each of three variables (PChE serum, PChE liquor, and PChE CSF/serum ratio) to determine the optimal cut-off point that maximized the desired test properties. The optimal cut-off of the investigated parameters was calculated as the threshold value with the highest specificity and sensitivity. For the cut-off points, results are presented as sensitivity, specificity, and positive and negative predictive values (PPV and NPV). For each variable, area under the curve (AUC) of sensitivity plotted against 1-specificity is also reported with 95% confidence intervals (CI).

## Results

Groups did not significantly differ by sex (*P* = 0.795), but they did by age (*P* = 0.001). In the study group, there were 12 girls and 18 boys between the ages of 2.8 and 16 years (median 8.38 years; IQR 4-11.25). In the control group there were 16 boys and 14 girls between the ages of 2 months and 16 years (3 years; IQR 1.08-6.54).

PChE activity in CSF showed a positive correlation with age in the study group (*P* = 0.037, r = 0.38) and a negative correlation in the control group (*P* = 0.883, r = -0.03). It was the same in the serum (study group, *P* = 0.057, r = 0.35; control group, *P* =0.259, r = - 0.21). PChE CSF/serum ratio showed a positive correlation with age in both study (*P* = 0.384, r = 0165) and control group (*P* = 0.74, r = 0.063).

**Figure 1:**
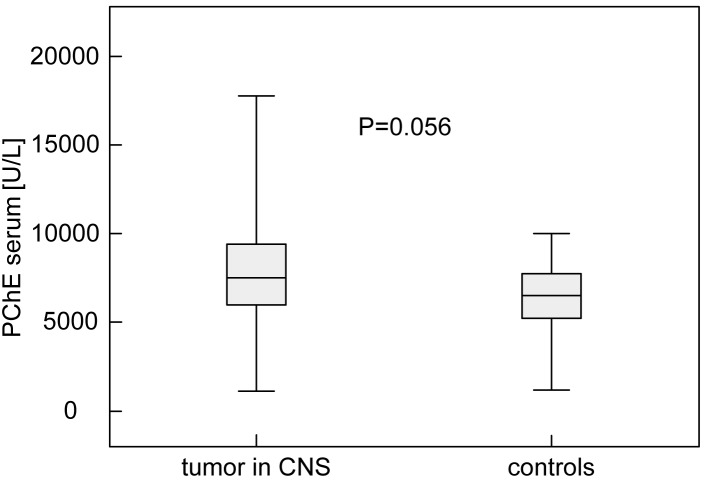
Pseudocholinesterase (PChE) activity in serum (U/L) in children with solid central nervous system (CNS) tumors and control group

**Figure 2:**
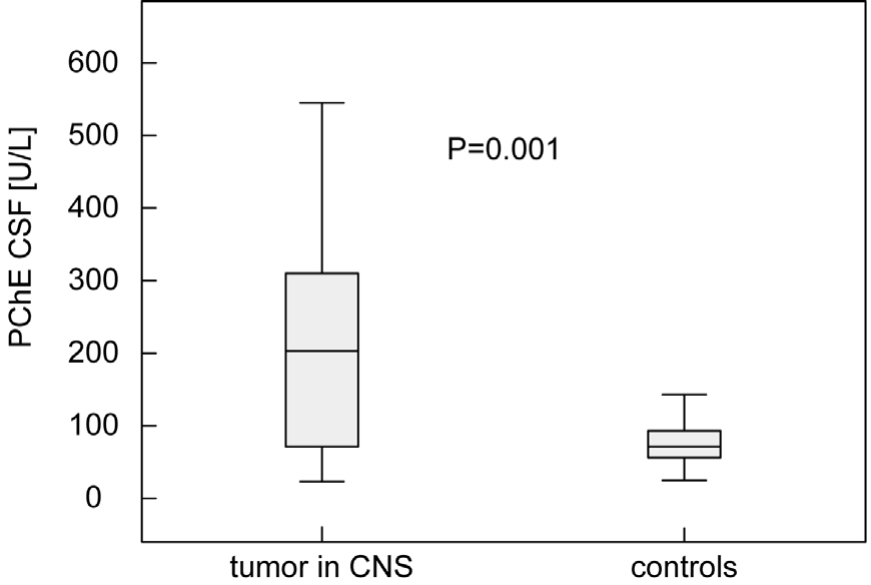
Pseudocholinesterase (PChE) activity in cerebrospinal fluid (CSF) (U/L) in children with solid central nervous system (CNS) tumors and control group

**Figure 3:**
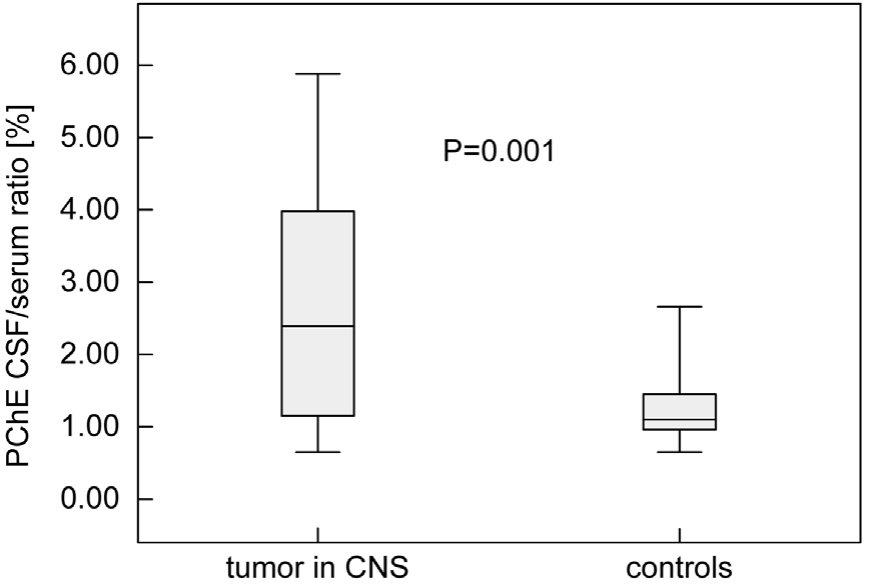
Pseudocholinesterase (PChE) cerebrospinal fluid (CSF)/serum ratio (%) in children with solid central nervous system (CNS) tumors and control group

In the control group, the median PChE activity in the serum was 6488 U/L (IQR, 5202-7772) and in CSF 70 U/L (IQR, 55-93) (Figure 1,2). Median PChE CSF/serum ratio was 1.09% (IQR, 0.95-1.45) and the peak PChE CSF/serum ratio was 2.66% (Figure 3). In the study group, the median PChE activity in serum was 7505 U/L (IQR 5972-9397) and 202 U/L (IQR 70-310) in CSF (Figure 1,2). The median PChE CSF/serum ratio was 2.38% (IQR 1.14-3.97) and the peak PChE CSF/serum ratio was 5.86% (Figure 3). PChE activity in CSF as well as PChE CSF/serum ratio were significantly higher in the study than in the control group (PChE in CSF, *P* = 0.001; PChE CSF/serum ratio, *P* = 0.001). PChE activity in the serum was higher in the study group, but not significantly (*P* = 0.056).

The results indicate high variability of PChE activity in CSF in the study group. In this group, we also found a positive correlation (r = 0.68) between PChE activity in the serum and PChE CSF/serum ratio (*P* = 0.001). In the control group, this correlation was also positive (r = 0.26) but not significant (*P* = 0.167).

**Figure 4:**
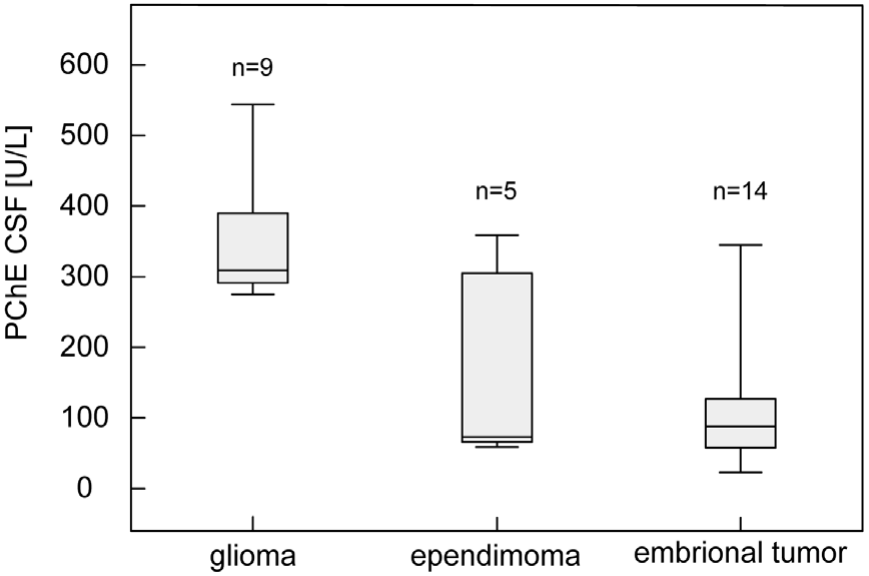
Pseudocholinesterase (PChE) activity in cerebrospinal fluid (CSF) (U/L) in various types of solid central nervous system (CNS) tumors in children

**Figure 5:**
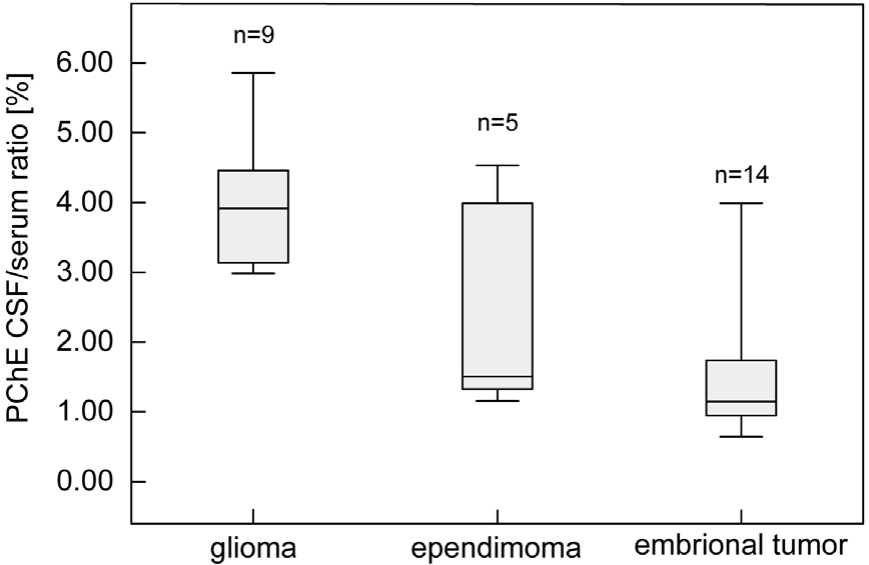
Pseudocholinesterase (PChE) cerebrospinal fluid (CSF)/serum ratio (%) in various types of solid central nervous system (CNS) tumors in children

Nine children in the study group had gliomas. In this group, the PChE CSF/serum ratio variable was between 3 and 5.86%, and the differences from the control group were the most pronounced. PChE CSF/serum ratio and PChE in CSF in other tumors were lower than in gliomas (Figure 4,5).

**Figure 6:**
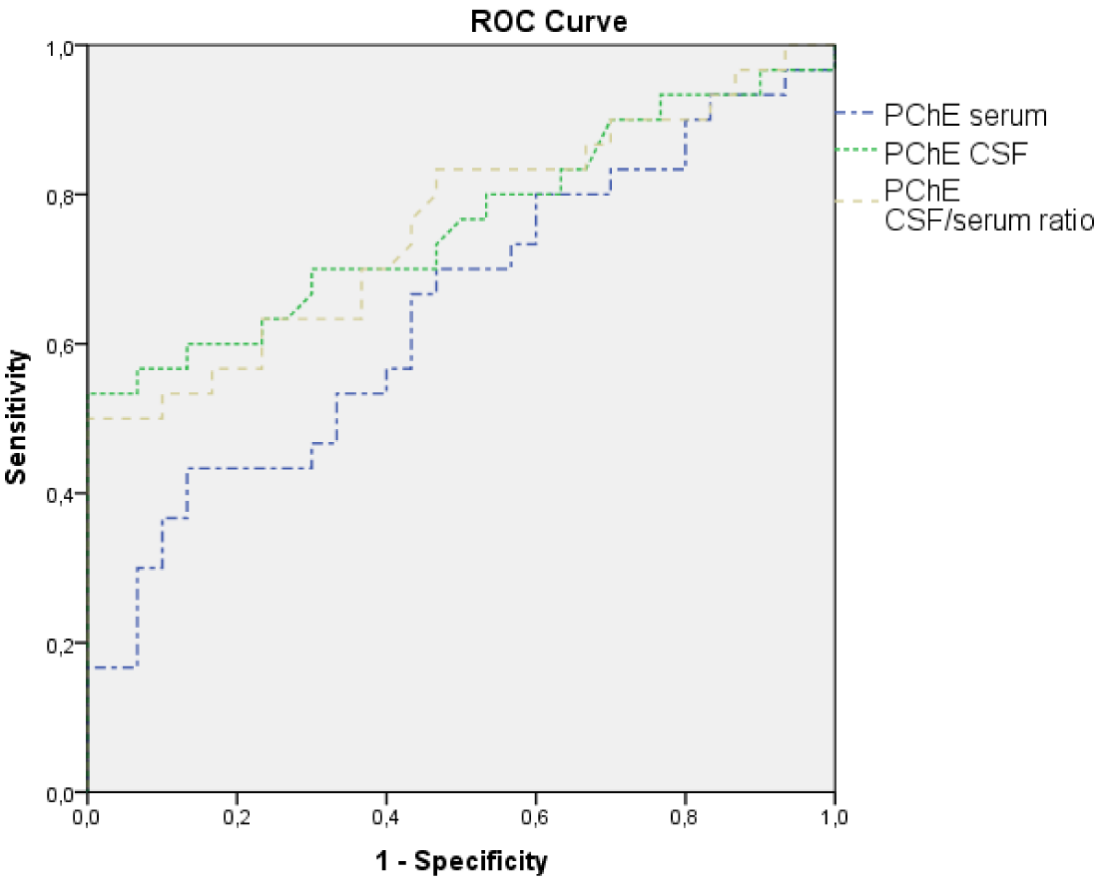
Receiver operating characteristic (ROC) curves for predicting solid central nervous system (CNS) tumor in children based on pseudocholinesterase (PChE) activity in serum, cerebrospinal fluid (CSF), and PChE CSF/serum ratio

Using a ROC curve analysis we tested PChE in CSF and serum and PChE CSF/serum ratio to determine whether it was a suitable variable for identifying the presence of tumors in CNS/CSF. For serum PChE, the AUC was 0.64 (95% CI 0.50-0.78), for CSF PChE 0.65 (95% CI 0.55-0.75), and for PChE CSF/serum ratio 0.76 (95% CI 0.63-0.88). ROC analysis suggested that the CSF PChE cut-off of 80 U/L (21/30 patients with tumors had PChE in CSF>80 U/L) could produce 70% sensitivity, 70% specificity, 70% PPV, and 70% NPV. When the cut-off value was further increased to 150 U/L, all 16 patients with PChE CSF levels >150 U/L were positive for solid tumor, corresponding to a specificity of 100%, sensitivity of 53%, PPV of 100%, and NPV of 68%. For PChE CSF/serum ratio, a cut-off of 1.09 was evaluated. Twenty-five of 29 patients with elevated PChE CSF/serum ratio were positive for tumor, corresponding to a sensitivity of 83%, specificity of 53%, PPV of 64%, and NPV of 76%. In order to maximize specificity, a cut-off of 2.70% was introduced, corresponding to a specificity of 100%, sensitivity of 50%, PPV of 100%, and NPV of 67% (Figure 6).

All children in both groups had CSF cytological, biochemical, and microbiological test results within the reference range according their age. Their total protein and albumin values were also within the reference range. This confirmed that other clinical conditions affecting CSF were not present in these children.

We registered all drugs taken by children in both groups at the time of sampling. In the control group, 21 children received no therapy and 9 received medication. Three children received ranitidine, 2 valproic acid, 1 child promazine sodium, 1 cefuroxime, 1 prednisone, and 1 phenobarbitone, nitrazepam, pantoprazole, vitamin D3, lactulose, and vigabatrin. In the study group, 18 children received no therapy. Seven children received ranitidine, 1 hydrocortisone, 1 acyclovir, 1 child fluconazole, 1 trimetoprim sulfometoxazole, and 1 radiotherapy.

## Discussion

Our research was first to analyze PChE activity in CSF in children with solid CNS tumors and it showed that they had higher PChE activity in CSF than children who did not have tumors. Our aim was to find a relatively easily available biomarker for solid CNS tumors in children that would require less invasive methods. Tissue samples from the CNS are mainly unavailable, which is why we chose to test CSF, which is in direct contact with the brain structures. Around 528 proteins secreted by a tumor have been identified in CSF to date (17), many of which are studied as potential biomarkers (3,17).

There are several limitations of this study. Research was conducted in the Children’s Hospital Zagreb, which is the Reference Centre for Solid Tumors in Children of the Ministry of Health and Social Welfare in Croatia. Due to the size of Croatian population, collecting a larger number of examinees would require considerably more time. For this reason, different types of solid CNS tumors were included in the study. Also groups differed by age, although it is known that PChE activity is different in different age groups (14). Considering the age of our participants, we expected PChE activity to be higher in the control group because of their younger age (14). Still, PChE activity was significantly higher in the study group. Correlation of age and PChE activity in CSF and serum was positive in the case group, ie, older children had greater PChE activity in both serum and CSF. This further supports the assumption that PChE activity was indeed higher due to the presence of tumors.

Participants in both groups received drugs that could have affected PChE activity. Antiepileptic drugs or sedatives are acting as AChE and PChE inductors in the serum (18,19), while ranitidine and hydrocortisone lower PChE activity (17,20,21). Their effect diminished the differences between the groups rather than augmented them, as was expected. PChE activity in CSF and serum was much higher in the study group and maybe it would have been higher had it not been affected by hydrocortisone and ranitidine. In the control group, PChE activity maybe would have been lower had they not taken antiepileptic therapy.

The variability of PChE activity in CSF was higher in children with tumors. We believe that this is a result of differences between tumor types, especially in relation to glioma tissue. Cavanagh et al showed that glioma tissue had high PChE concentration levels (4). In our study, patients with gliomas also had the highest PChE activity values. These differences require further study. To rule out any influence of the physiology and pathology of the examinees, we calculated CSF/serum PChE ratio, which was significantly higher in the study group. In conclusion, this study indicates that PChE activity in CSF expressed as a CSF/serum PChE ratio can be used to detect solid CNS tumors in children.
